# Subdural Empyema as a Sequela of Severe Erosive Sinusitis: A Case Report

**DOI:** 10.7759/cureus.67690

**Published:** 2024-08-24

**Authors:** Ahmed Madan, Madison Wilson, Andy Garcia, Feisal Hamam, Ravindar Rhandhawa

**Affiliations:** 1 General Surgery, Larkin Community Hospital, South Miami, USA; 2 Biomedical Sciences, Barry University, Miami, USA; 3 General Surgery, St. George's University School of Medicine, St. George's, GRD; 4 Emergency Medicine, St. George's University School of Medicine, St. George's, GRD; 5 Otolaryngology, Delray Medical Center, Delray Beach, USA

**Keywords:** functional endoscopic sinus surgery, infectious, craniotomy, sinusitis, subdural empyema

## Abstract

Intracranial subdural empyema is a rare but critical neurosurgical emergency marked by pus accumulation between the brain and the dura mater. It typically arises from bacterial or fungal infections, often secondary to sinusitis, otitis media, or head trauma. Symptoms can range from mild headaches to significant neurological deficits and altered mental status. Diagnosis is confirmed through advanced imaging techniques such as MRI and CT scans. Timely intervention is essential to prevent neurological damage and systemic complications, usually involving surgical drainage and antimicrobial therapy. We present the case of a 45-year-old male who visited the emergency room several times with progressive lethargy and altered mental status. He was admitted and later transferred to our trauma center for a suspected subdural hematoma. An emergent right-sided craniotomy was performed, and a subdural empyema was found. The patient improved following subdural drainage and antibiotic treatment, including 600 mg linezolid every 12 hours, 2 g cefepime every eight hours, and 500 mg metronidazole every eight hours. This case highlights the effectiveness of prompt medical and surgical intervention in managing this rare condition and offers valuable insights for improving future patient outcomes.

## Introduction

Intracranial subdural empyema is a rare and life-threatening pyogenic infection localized between the dura and arachnoid mater, constituting a neurosurgical emergency [1]. This condition typically arises when a bacterial or fungal infection extends into the intracranial spaces, often following complications such as immunodeficiency, chronic sinusitis, otitis media, mastoiditis, neurosurgical procedures, or head injuries [2]. Given its severity, urgent identification and intervention are crucial to reduce the risks of neurological impairment and systemic complications.

The diagnosis of intracranial subdural empyema is challenging due to its nonspecific presentation and rapid progression. Clinically, it often presents with headaches, fever, and altered mental status. As the condition progresses, more severe symptoms such as focal neurological deficits, seizures, meningeal irritation, cerebritis, and increased intracranial pressure may develop [3]. We present a case where an intracranial subdural empyema resulted from complex sinusitis. The rarity of this condition and the limited literature available necessitate detailed documentation of our patient’s case to facilitate prompt recognition and differentiation from other similar conditions and to illustrate the effective medical and surgical treatments administered by our medical team.

## Case presentation

**Figure 1:**
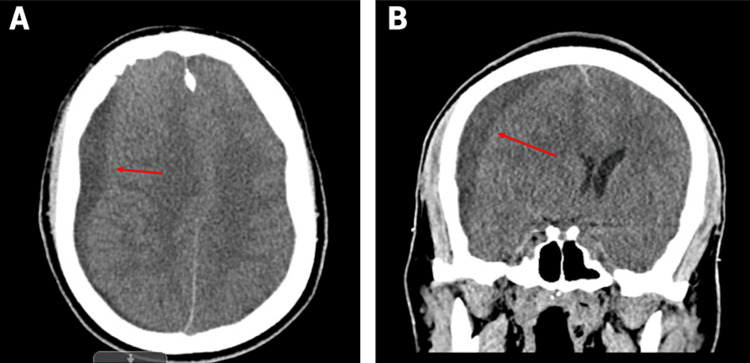
Initial CT imaging showing subdural empyema: (A) transverse view and (B) coronal view

A 45-year-old male with altered mental status and unresponsiveness was transferred to our Level One trauma center for neurosurgical evaluation after brain CT imaging revealed a right-sided subdural hematoma and a 12.5 mm midline shift, as shown in Figure 1. Prior to his arrival at our center, the patient had exhibited a three-week history of progressive lethargy and deconditioning but had no previous history of trauma. He had visited another emergency room twice with these symptoms and was evaluated and discharged on both occasions before presenting again with severe unresponsiveness. Upon arrival at our trauma bay, the patient was intubated, mechanically ventilated, and unresponsive, with a Glasgow Coma Scale (GCS) score of 5T. Primary and secondary surveys were conducted, and previous CT imaging was intensively reviewed. Given the information, an urgent decision was made to proceed with an emergency right-sided craniotomy, involving approximately one-third of the superior temporal line via a cruciate incision and endoscopic evacuation of the hematoma.

Preoperatively, the patient was administered 2 g of Ancef. During the craniotomy, once the portion of the skull was removed, the subdural membrane exhibited an atypical yellow discoloration. Upon opening the dura, purulent fluid, consistent with subdural empyema, was expelled under pressure. The pus was evacuated, and the fluid was sent for culture. The discoloration was also noted on the arachnoid tissue, along with subarachnoid purulence. Substantial irrigation with polymyxin was performed until clear fluid returned. A 7 mm flat JP drain was placed in the subdural compartment before reapproximating the dura, bone flap, temporalis muscle, and dermis. Postoperatively, CT imaging confirmed a small residual subdural collection measuring 2-3 mm, a reduction in the midline shift from 10 mm to 3 mm, and improved expansion of the right ventricular system, though bilateral paranasal sinuses remained opacified. The postoperative workup revealed a significantly elevated white blood cell count of 22.8 × 10³/µL, indicating an infectious etiology rather than the original hemorrhage theory.

**Figure 2:**
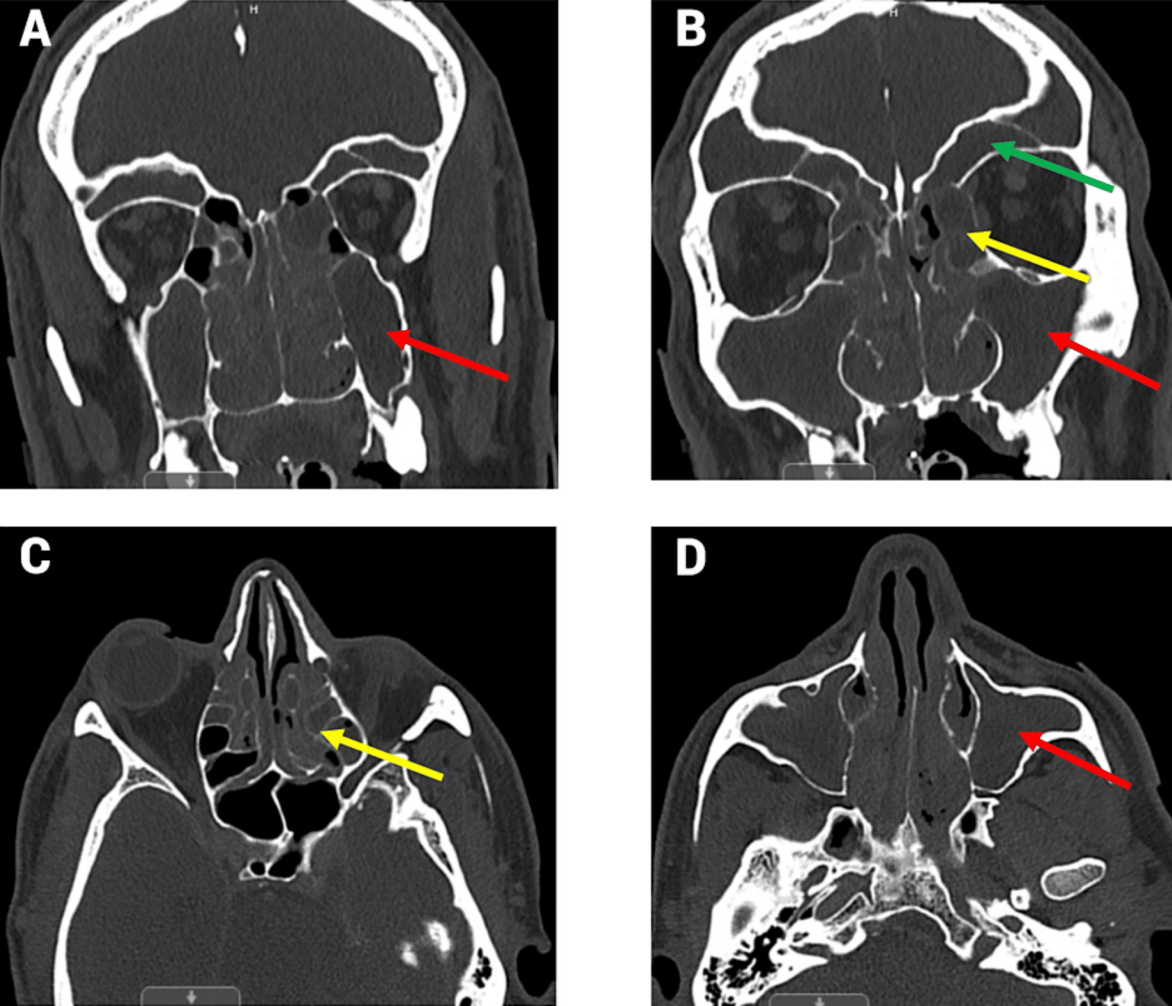
(A, B) Coronal CT images and (C, D) transverse CT images showing extensive sinusitis in the frontal sinuses (green arrow), maxillary sinuses (red arrow), and ethmoid air cells (yellow arrow)

**Figure 3:**
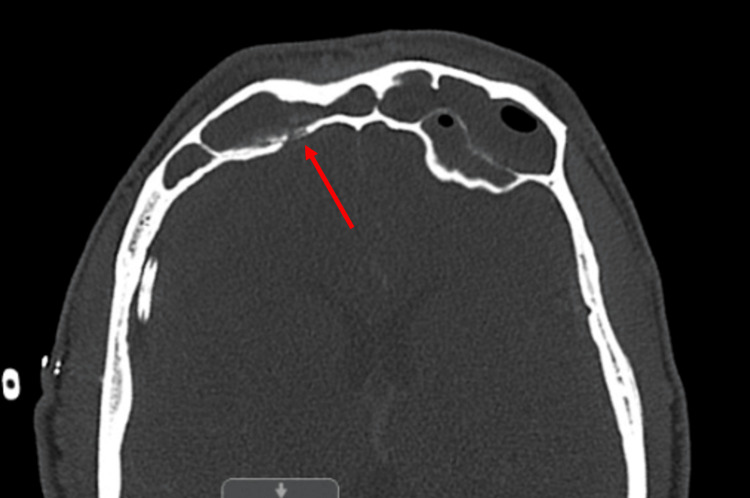
Demineralization of the right frontal sinus posterior wall, indicated by the arrow

**Figure 4:**
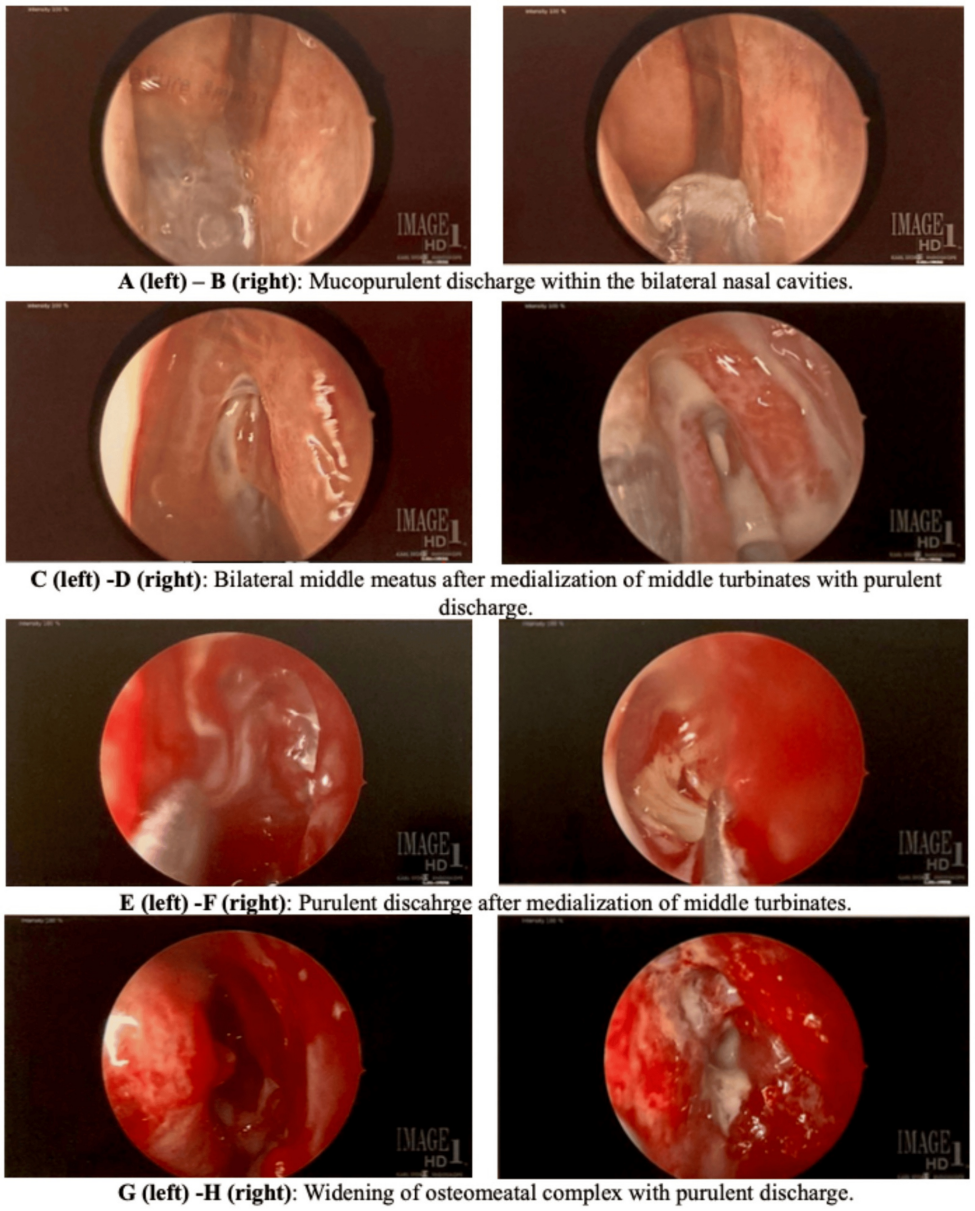
(A-H) Endoscopic images of the bilateral nasal cavities during FESS FESS, functional endoscopic sinus surgery

Given these findings, a CT of the sinuses with contrast was performed, revealing extensive sinusitis of the frontal, maxillary, and ethmoid air cells, as shown in Figure 2. Demineralization of the right frontal sinus posterior wall, observed in Figure 3, was thought to be the likely entry point for the subdural empyema. An MRI of the sinuses was attempted but was suboptimal due to the patient’s lack of cooperation. The subdural fluid culture tested positive for gram-positive cocci. Infectious disease was consulted, and a regimen of 600 mg linezolid every 12 hours, 2 g cefepime every eight hours, and 500 mg Flagyl every eight hours was recommended. Given the CT results, otorhinolaryngology was also consulted. The otorhinolaryngologist planned functional endoscopic sinus surgery (FESS) and added a course of 4 mg dexamethasone every eight hours to reduce mucosal inflammation and facilitate sinus drainage prior to the procedure. During FESS, purulent secretions were cultured and suctioned from the bilateral frontal and maxillary sinuses and the right ethmoid sinus, as shown in Figure 4. The left ethmoid sinus was clear on CT imaging and was not opened. Copious irrigation was provided to the affected paranasal sinuses before concluding the procedure. Cultures from the sinuses were positive for *Enterobacter aerogenes*.

Four days after the balloon sinuplasty and sinus irrigation, repeat imaging revealed reaccumulation in the subdural space, although the sinuses had cleared. Given this, it was decided that the patient would benefit from additional drainage. The patient was taken back to the operating room, where a successful drain placement was performed without postoperative complications. The patient subsequently improved to a GCS score of 15. Following discharge to a skilled nursing facility, the patient continued on 600 mg linezolid every 12 hours and 2 g cefepime every eight hours for an additional three weeks.

## Discussion

Sinusitis is a common, manageable infection that rarely progresses to severe complications, with such cases occurring in less than 1 of every 1000 presentations. Of these rare complications, around 80% are orbitocranial, while the remainder spread to the intracranial cavities [4]. Specifically, subdural empyema, a severe form of sinusitis complication, has a significantly higher mortality rate of up to 28% compared to other complications [5]. Although subdural empyema can initially present with nonspecific symptoms such as nausea, vomiting, and headaches, it may progress to neurological deficits if left untreated [6]. The severity of intracranial subdural empyema is due to the continuous subdural space, which lacks anatomical barriers and allows the empyema to spread throughout the brain, potentially reaching both cerebral hemispheres and the posterior fossa [7]. In our patient, the nonspecific symptoms of headaches and sinus congestion seen on CT were initially mistaken for minor issues but rapidly progressed to a life-threatening condition.

The pathophysiology of intracranial empyema can occur via direct or indirect pathways. The indirect route involves an infection from the ear or paranasal sinuses that drains into the intracranial abscess through valveless emissary veins [8]. The less common direct route requires either a congenital malformation or bone erosion into the intracranial cavity [9]. In our patient, the demineralization of the right frontal sinus posterior wall facilitated bacterial spread into the subdural space, leading to empyema formation. The pathogens typically associated with paranasal sinus infections that cause intracranial subdural empyema include aerobic, anaerobic, and microaerophilic organisms [10], with *Staphylococcus aureus* being a common cause in post-traumatic cases [11]. Our patient’s polymicrobial culture necessitated consultation and management by infectious disease specialists, who prescribed linezolid, cefepime, and Flagyl.

The advent of antibiotics has significantly reduced the mortality rate for subdural empyema, from nearly 100% in the pre-antibiotic era to 14-28% today [2]. Studies comparing treatments for bacterial brain abscesses have shown that meropenem monotherapy may be more effective than the standard combination of intravenous cefotaxime and metronidazole [12]. Consequently, we relied on our infectious disease team to manage the patient’s antibiotic therapy. Prompt CT imaging remains crucial for diagnosing subdural empyema, as evidenced by our patient’s initial CT suggesting a subdural hematoma before identifying the empyema in the operating room. This highlights the importance of considering and ruling out differential diagnoses. The established management for subdural empyema - rapid surgical drainage and antibiotic therapy - was effective in treating our patient.

## Conclusions

Subdural empyemas often present with nonspecific symptoms, making timely diagnosis challenging. As the infection progresses, it can rapidly escalate into a life-threatening emergency. Fortunately, this patient’s condition was diagnosed and treated within the critical survival window at our center, and the patient continues to show daily improvement. Prompt identification and treatment of intracranial empyema are crucial for survival; thus, medical professionals must be adept at recognizing this complication and ruling it out when clinical suspicion arises.
